# Mouse models of atherosclerosis: a historical perspective and recent advances

**DOI:** 10.1186/s12944-016-0402-5

**Published:** 2017-01-17

**Authors:** Yee Ting Lee, Hiu Yu Lin, Yin Wah Fiona Chan, Ka Hou Christien Li, Olivia Tsz Ling To, Bryan P Yan, Tong Liu, Guangping Li, Wing Tak Wong, Wendy Keung, Gary Tse

**Affiliations:** 1 School of Biomedical Sciences, Li Ka Shing Faculty of Medicine, University of Hong Kong, Hong Kong, SAR People’s Republic of China; 2 School of Biological Sciences, University of Cambridge, Cambridge, UK; 3 Faculty of Medicine, Newcastle University, Newcastle, UK; 4 Department of Medicine and Therapeutics, Chinese University of Hong Kong, Hong Kong, SAR People’s Republic of China; 5 Department of Epidemiology and Preventive Medicine, Monash University, Melbourne, Australia; 6 Tianjin Key Laboratory of Ionic-Molecular Function of Cardiovascular disease, Department of Cardiology, Tianjin Institute of Cardiology, Second Hospital of Tianjin Medical University, Tianjin, 300211 People’s Republic of China; 7 School of Life Sciences, Chinese University of Hong Kong, Hong Kong, SAR People’s Republic of China; 8 Stem Cell & Regenerative Medicine Consortium, Li Ka Shing Faculty of Medicine, The University of Hong Kong, Pokfulam, Hong Kong, SAR People’s Republic of China; 9 Li Ka Shing Institute of Health Sciences, Chinese University of Hong Kong, Hong Kong, SAR People’s Republic of China

**Keywords:** Mouse models, Atherosclerosis, ApoE, LDL receptor, Reactive oxygen species, ER stress, Mitochondrial dysfunction

## Abstract

Atherosclerosis represents a significant cause of morbidity and mortality in both the developed and developing countries. Animal models of atherosclerosis have served as valuable tools for providing insights on its aetiology, pathophysiology and complications. They can be used for invasive interrogation of physiological function and provide a platform for testing the efficacy and safety of different pharmacological therapies. Compared to studies using human subjects, animal models have the advantages of being easier to manage, with controllable diet and environmental risk factors. Moreover, pathophysiological changes can be induced either genetically or pharmacologically to study the harmful effects of these interventions. There is no single ideal animal model, as different systems are suitable for different research objectives. A good understanding of the similarities and differences to humans enables effective extrapolation of data for translational application. In this article, we will examine the different mouse models for the study and elucidation of the pathophysiological mechanisms underlying atherosclerosis. We also review recent advances in the field, such as the role of oxidative stress in promoting endoplasmic reticulum stress, mitochondrial dysfunction and mitochondrial DNA damage, which can result in vascular inflammation and atherosclerosis. Finally, novel therapeutic approaches to reduce vascular damage caused by chronic inflammation using microRNA and nano-medicine technology, are discussed.

## Background

Atherosclerosis is responsible for acute myocardial infarction and cerebrovascular accidents, accounting for the majority of cardiovascular deaths. It is a chronic inflammatory disease characterized by the development of complex atherosclerotic plaques, leading to hardening and narrowing of the arterial lumen. Atherosclerotic plaque formation is initiated and sustained by a combination of endothelium dysfunction and chronic exposure to cardiovascular risk factors that promote vascular inflammation, such as hyperlipidemia, hypertension, smoking, male gender and diabetes.

Among these, the single most important risk factor is high plasma low density lipoprotein (LDL) level, of which a monogenetic cause is familial hypercholesterolemia [1]. Other risk factors are important in individuals with normal LDL levels. In the presence of a LDL level permissive of atherosclerotic plaque formation, these risk factors are important in explaining the development and progression of atherosclerotic lesions [2, 3]. Conversely, these are less important in individuals with very low LDL levels, who are unlikely to develop atherosclerosis irrespective of other risk factors [4]. With this in mind, cholesterol loading in the lesion-containing foam cells has been the focal point of intense research and has been studied extensively using different animal models. The extent of cholesterol loading is the result of lipoprotein uptake and lipid efflux in foam cells has been targeted therapeutically to reduce lesion size [5].

### LDL accumulation

Excess LDL in plasma accumulate in the sub-endothelial space of arterial wall and undergoes oxidation to become oxidized LDL (oxLDL) [6]. This then triggers an inflammatory response and induces the expression of chemotactic protein such as vascular cell adhesion molecule-1 (VCAM-1), E-selectin and P-selectin in the endothelium [7, 8]. The expression of adhesion molecules attracts blood cells into the injured arterial wall, with monocytes being the most prominent cell type [9]. Upon migration into the intimal layer, monocytes differentiate into macrophages which avidly internalize oxLDL and become foam cells themselves [10].

Foam cells present antigens to immune cells, such as circulating monocytes and T-cells, whose activation contributes critically to plaque development [11]. They also secrete mediators to perpetuate the inflammatory process in the arterial wall and to stimulate migration of smooth muscle cells from the tunica media into sub-endothelial space. Mediated by platelet derived growth factor, smooth muscle cells exhibit abnormally high proliferation rates and secrete extracellular matrix proteins that contribute to fibrous cap formation [12, 13]. The fibrous cap protects the core of the plaque from circulating blood cells, especially platelets that are responsible for the thrombosis associated with plaque rupture. This maladaptive response of a non-resolving inflammation is the driving force of atherosclerotic plaque development. It is worth noting that the SMCs from different regions of the microvasculature have different developmental origins [14], which can contribute to site-specific atherosclerosis responses [15].

During plaque evolution, macrophages proliferate and undergo apoptosis and efferocytosis [16, 17]. Depending on the efficiency of efferocytosis, apoptotic cells may be removed, leading to reduction of lesion size [18], or they may accumulate and be subjected to secondary necrosis, producing a necrotic core characteristic of advanced plaques. The accumulation of apoptotic bodies will enhance the plaque instability by triggering inflammation [19, 20].

Whilst foam cells are the most abundant leukocytes within the atherosclerotic lesion, studies in mouse models have implicated other cell types, including neutrophils, T-and B-cell, and mast cells, in atherogenesis [21, 22]. These cells, though contribute little to the lesion mass, can influence atherosclerosis by secreting variety of proteins that regulate other cells or components within the plaque. Experiment studies using mice have demonstrated that among the subsets of T-cells, Th1 cells and natural killer T-cells are notably pro-atherogenic, whilst the role of Th2 and Th17 cells are less well understood [23–25].

### Plaque rupture

Plaque rupture is responsible for the adverse consequences of ischemic events in acute myocardial infarction and cerebrovascular accidents and death [26]. At advanced stages of atherosclerosis, rupture of vulnerable plaques exposes their thrombogenic compounds, thereby leading to luminal thrombosis [27]. Macrophage-derived proteases, especially metalloproteases, can destabilize plaques, but the exact underlying mechanism of plaque vulnerability remained incompletely elucidated [28].

## Animal models of atherosclerosis

The first key piece of evidence that atherosclerosis is inducible in laboratory animals was provided by Ignatowski in 1908, who demonstrated lesion formation in the aortic wall of rabbits that were fed a diet enriched in animal protein (mainly meat, milk, and egg yolk). Since then, various animal species, such as rabbits, mice, rats, guinea pigs, hamsters, birds, dogs and non-human primates, have been used for experimentation. Of these, rodents, swine and rabbits have provided crucial information for the pathophysiological mechanisms underlying the initiation and subsequent development of atherosclerotic plaques. In spite of the many differences between the animal models, all of these demonstrate the requirement of high blood cholesterol levels for plaque development. The observations of this remarkable characteristic supported the discovery of the essential role of cholesterol in atherosclerosis development. Animal models have been extensively used for the study of human cardiovascular diseases [29–40], and their use has led to opportunities for translational application [32, 41–45]. In this review, the different models for examining atherosclerosis, their own advantages and disadvantages (Table 1) and the molecular pathways involved (Table 2), will be discussed in turn.

**Table 1:** Advantages and disadvantages of mouse models for studying atherosclerosis

Advantages	Disadvantages
Ease of genetic manipulation	Toxic diet needed to induce atherosclerosis in wild-type mice
Low maintenance cost	Different lipid metabolism compared to humans: high HDL, no CETP
Short generation time means less time consuming for research projects	Differing cardiovascular anatomy and physiology with different predisposed site for the development atherosclerosis
Wide availability	Small body size limits frequent blood collection and increases difficulty of dissection of small arteries

**Table 2:** Molecular pathways involved in atherosclerosis

Molecular mechanisms	Role in Atherosclerosis	References
Expression of vascular cell adhesion molecule-1 (VCAM-1), E-selectin and P-selectin	Inflammatory response induced by LDL oxidation	[7, 8]
Nuclear factor-kappa B (NF-κB) activation	Chemotaxis via regulation of chemokines, such as CCL2, CCL5, CCL8, and CXCL9	[166, 169]
Release of platelet derived growth factor	Fibrous cap formation	[12, 13]
CHOP activation	Macrophage apoptosis via endoplasmic reticulum stress	[107, 108]
Pattern recognition receptor activation	Macrophage apoptosis via activation of CD36-TLR2 pathway	[111, 112]
Activation of mitochondrial, Ca^2+^-dependent pathways	Vascular smooth muscle cell apoptosis via calpain/mPTP/cytochrome C/caspase-3 and apoptosis-inducing factor	[140]
cytochrome c release and activation of caspase-9 and the effector caspases	Macrophage apoptosis induced by cholesterol loading	[141]
Toll-like receptor activation	Immune activation through recognition of mitochondrial DNA, which can act as damage-associated molecular patterns (DAMPs)	[148]
Upregulation of transient receptor potential cation (TRPC) channels	VSMC, migration, proliferation and apoptosis; neointimal proliferation	[158, 160–162]

### Mouse

With its small size and ease of genetic manipulation, the mouse is currently the most frequently used model in atherosclerosis research [46]. Development of atherosclerosis is influenced by a number of genes, which interact with each other and the environment to affect the disease phenotype [47]. Manipulation can take place for single genes, and indeed in a time-dependent manner, which permits the elucidation of the molecular mechanisms operating in different phase of plaque evolution, whilst the manipulation of gene expression in specific cell types has increased our understanding of the contributions of different immune cells to atherosclerosis.

#### Dietary models

The first mouse model was developed by Wissler and coworkers in 1960s [48]. They fed the mice with a diet of high fat 30%, high cholesterol 5% and cholic acid 2%. This diet successfully promoted hypercholesterolemia, and induced the formation of fatty streaks in different vascular regions [49]. However, the high fat diet induced a high level of acute inflammatory changes, but did not simulate the chronic low-grade inflammatory environment observed in human atherosclerosis [50]. Also, the pro-inflammatory diet as highly toxic, which led to weight loss and increased the susceptibility of the animals to infections.

A less toxic diet of 15% fat, 1.25% cholesterol and 0.5% cholic acid was introduced, resulting in high variability of diet-induced atherosclerosis in different strains of mice [51]. The most susceptible strain was C57BL/6, which developed mild hypercholesterolemia — around 200 mg/ml — and fatty streak lesions in the aortic root after 3–9 months of fat feeding [52]. However, the lesion was not comparable to that observed in humans, with small lesions found in the aorta, consisted almost exclusively of macrophages and did not progress to fibrous plaques. The difference in morphology of the plaque formation between mouse and human hampers data extrapolation. Nevertheless, from experiments conducted in dietary models, a number of genetic loci that increase the susceptibility to atherosclerosis has been identified. An example is Ath-1, which was mapped to the short arm of chromosome 1 [53].

Recent experiments in germ-free mice generated by feeding with an antibiotic cocktail have demonstrated dietary lipid phosphatidylcholine (lecithin) was identified by a metabolomics approach, and these were subsequently shown to be predictive of atherosclerosis in humans, highlighting the role of intestinal microbiome in regulating plasma lipoprotein homeostasis [54].

#### ApoE knockout and LDLR-deficient models

Given the toxicity of high fat diet and differences in lesion morphology, limitations of using wild-type mice for studying atherosclerosis were recognized. The development of genetically modified mice represented a breakthrough. Gene knockout and knock-in techniques, together with the ability to control the spatial and temporal patterns of gene expression, have proven useful for studying atherosclerosis. The first genetically modified mouse model developed was the Apolipoprotein E (ApoE) knockout model [55]. ApoE is a structural component of all lipoprotein except for LDL and is a critical ligand for the hepatic clearance of plasma lipoproteins mediated by LDLR and LDLR-related protein [56–58]. ApoE knockout mice exhibited significant hypercholesterolemia (400 mg/dl, which represents a five-fold increase in plasma cholesterol level compared to wild type mice) despite being on a low fat diet [59–61]. An advantage of using ApoE knockout mice is that the high toxic diet can be avoided. Moreover, it shares greater degrees of similarity in the atherosclerotic development to humans compared to wild-type models. There is dramatic shift in plasma lipoprotein profile, with the proatherogenic VLDL as the most abundant circulating lipoprotein, similar to type II hyperlipidemia in humans [62]. Spontaneous development of atherosclerotic lesions in several vascular beds, predominantly in the aortic root, aortic arch and different branch point along the aorta, are observed [63]. Another advantage is the rapid development of lesions compared to wild-type. When ApoE knockout mice are fed with a high fat diet [64, 65] (Western type diet, which contains 0.15% cholesterol, 21% fat derived from milk and without the use of cholic acid), they exhibit high plasma lipid level of over 1000 mg/dl and develop complex fibrous plaques in the aortic sinus after 10–14 weeks of diet.

However, ApoE knockout mice has its own drawbacks which limits the extrapolation of data derived from this model to humans. Firstly, the lipid metabolism is dissimilar, with the majority of the plasma cholesterol as VLDL but not LDL as observed in humans [66, 67]. Secondly, ApoE was found to provide extra athero-protective properties in addition to mediating lipoprotein clearance, by virtue of its anti-oxidative, anti-proliferative and anti-inflammatory actions [68]. Therefore, atherosclerosis in ApoE knockout model may be independent of plasma lipid levels. Conversely, low levels of ApoE expressed in adrenal cells can reduce the severity of atherosclerosis without affecting plasma lipid levels in ApoE-deficient mice [69].

To overcome the problems of the above model, LDLR deficient mouse model was developed. Compared to ApoE, LDLR has fewer functions and therefore the effects due to its absence are more easily attributed to lipoprotein homeostasis than other processes such as cell proliferation or inflammation [70]. LDLR deficiency impairs lipoprotein uptake and clearance, resulting in a greater preponderance of LDL as the cholesterol-carrying plasma lipoprotein. Compared to ApoE knockout mice, this model shows a milder plasma cholesterol increase of 250 mg/dl when fed with standard low fat diet and the elevated lipoprotein is mainly LDL [71]. Whilst on high fat diet, LDLR-deficient animals developed severe hypercholesterolemia of 900 mg/dl with accumulation of VLDL and LDL, as well as extensive atherosclerosis [72].

Compared with ApoE deficiency, the absence of functional LDLR in humans is more common and leads to familial hypercholesterolemia, greatly increasing the cardiovascular risk [73]. This model shares the characteristics observed in human familial hypercholesterolemia, including the lesions in aortic valves and the aortic root [74]. This model has been useful for examining the relationship between diabetes and atherosclerosis, which often co-exist [72]. It was found that LDLR deficient mice were more prone to obesity and insulin resistance than ApoE deficient mice.

However, the LDLR model is imperfect with its own shortcoming. Firstly, compared with the ApoE knockout mice, the LDLR deficient mouse model is more resistant to injury-induced neointimal formation [75]. Therefore, ApoE knockout mice remains the better alternative for investigating the molecular mechanisms underlying restenosis following angioplasty [76]. Secondly, limited lesions developed only in older LDLR deficient mice with standard diet [77, 78]. Consequently, high fat diets with different levels of cholesterol have been employed to induce more significant atherosclerotic changes [79]. With a variety of dietary cholesterol intake, this model cannot be standardized across models generated by different laboratories.

#### Advantages and disadvantages of mouse models

The advantages of using small sized animals over larger animals are lower costs to purchase, breed, feed and maintain. The use of antibodies and drugs for intervention becomes more affordable with its small size. Another advantage is its rapid growth rate and short generation time [80]: it takes 6 to 8 weeks for the mouse to reach sexual maturity and approximately an additional 3 weeks for gestation [81]. It is relatively easy to control multiple genes by interbreeding given the wide availability of inbred strains [82], or using genetic knockout techniques to elucidate the role of individual genes in atherosclerosis [83]. The ease of genetic manipulation comes in the well-defined genetic makeup and availability of inbred strains.

The major limitation of mouse models is their natural resistance to atherosclerosis for several reasons. Firstly, cholesterol metabolism and lipoprotein pattern are different from those of humans due to the absence of cholesteryl ester transfer protein (CETP) [84], a carrier that facilitates the transport of cholesteryl esters and triglycerides between different lipoproteins [85]. Thus, mice usually have a lower plasma cholesterol levels of 60–100 mg/dl compared to humans, with the high density lipoprotein being the major circulating lipoprotein [86]. This contrasts with the deleterious LDL, which is the predominant form found in plasma of humans [87]. Thus, genetically-modified mice have been used to induce hypercholesterolemia, although concerns have been raised because this may be non-physiological and the pathogenesis may differ from that of human atherosclerosis [88].

Although the comparatively smaller size of mice results in more convenient use for experimentation, it also limits many aspects of practical investigation. For example, the coronary arteries are too small for visualization. Moreover, blood collection can be difficult [89]. There is also considerable difference in the anatomy of the cardiovascular system between mice and humans. The arterial intima consists of only endothelium overlying the internal elastic lamina without smooth muscle cells or connective tissues found in humans [90]. Moreover, the tunica media is less thick and the vasa vasorum is absent in mice [91].

The morphology of atherosclerotic lesions observed in mice is different from that of humans, in that the thick fibrous cap is absent [92]. In mice, vasa vasorum is not needed since the thin layers of tunica intima and intima do not pose a significant barrier to oxygen diffusion [91]. The absence of the vasa vasorum explains the lack of ingrowth of neovessels into the base of the lesions. The neovessel is thought to be derived from the vasa vasorum, considered as an important entry path for immune cells and contributes to chronic inflammation and development of the necrotic core [93]. The predilection sites for atherosclerosis development in mouse models are the aortic sinus and innominate artery, whereas the coronary arteries are commonly affected in humans. Unstable plaque is rarely observed in mouse models, and it is therefore difficult to examine plaque rupture and overlying luminal thrombosis, which is the direct cause of acute ischemic events responsible for cardiovascular deaths, using these systems [94–96].

## Recent advances

### Oxidative stress, dysfunction of intracellular organelles and vascular inflammation

Recent research efforts have focused on elucidating the mechanisms by which dysfunction of two intracellular organelles, the endoplasmic reticulum (ER) and the mitochondrion of various cell types, promotes vascular inflammation and atherosclerosis.

#### ER stress

The ER is responsible for protein synthesis, proper folding, maturation and assembly prior to further processing by the Golgi apparatus [97]. The highly oxidative environment within the ER lumen facilitates the formation of tertiary and quaternary structures, aided by chaperone proteins and a high concentration of luminal Ca^2+^ to facilitate their interactions [98]. Abnormalities in these processes can lead to misfolding or unfolding of proteins, which then accumulate within the ER lumen. This would increase ER stress, thereby activating the unfolded protein response (UPR) [99]. The UPR is a normal adaptive and protective mechanism to reduce the rate of protein synthesis, increase folding ability of proteins and aid misfolded or unfolded protein to cellular degradation pathways [100].

Prolonged ER stress of different cell types involved in atherosclerosis, including macrophages, VSMCs and endothelial cells, has been observed in atherosclerosis [101–103]. In macrophages, LDL and cholesterol are transported from late endosomes to the ER, where esterification of cholesterol and formation of lipid droplets occur [104]. In macrophages of atherosclerotic plaques, the esterification process is greatly reduced, which is responsible for cholesterol accumulation in foam cells [105]. It has been shown that UPR activation of macrophages takes place during the different stages of atherosclerotic lesion development in ApoE knockout mice [106]. Prolonged ER stress leads to macrophage apoptosis associated with expression of the UPR sensor C/EBPα-homologous protein (CHOP). CHOP inactivation could reduce the rate of macrophage apoptosis and plaque necrosis, suggesting that targeting of the UPR has the potential to hinder the progression of atherosclerosis [107, 108]. In early plaques, macrophages are efficient in clearing cells which have undergone apoptosis [109]. However, in advanced plaques, they are unable to do so [110], resulting in the formation of necrotic core [111]. Other mechanisms contributing to apoptosis of macrophages require activation of pattern recognition receptors (PRRs) of the innate immune system by oxidized lipids [111, 112]. PRR activation induces apoptosis via the CD36-TLR2 pathway [112]. In this pathway, the enzyme NADPH oxidase plays a key role in mediating oxidative stress, activation of the double-stranded RNA-dependent protein kinase, which induces CHOP and apoptosis [113].

ER stress in endothelial cells can be activated by shear stress [114, 115] or oxLDL [116]. This results in increased oxidative stress and therefore increased oxidation of sarcoplasmic/endoplasmic reticulum Ca^2+^-dependent ATPase, leading to atherogenesis [116]. In VSMCs, a number of cellular stressors, such as cholesterol loading, can initiate ER stress [117, 118], thereby activating apoptosis [119]. This may then affect collagen synthesis adversely. Homocysteine, which is raised in hypertension and diabetes, can initiate UPR [117]. ER stress plays a role in cardio-metabolic disorders such as hypertension and diabetes, and partly explains why atherosclerosis is worsened in the presence of these co-morbid conditions [120–122]. The use of mouse models has discovered novel mechanisms by which traditional pharmacological agents act to exert their vascular protective effects through modifying ER stress response [122–124]. For example, recent experiments have demonstrated that metformin, a commonly used anti-diabetic drug, protected endothelial function in obese, diabetic mice by inhibiting ER stress [120].

#### Mitochondrial dysfunction

The mitochondria are the cellular powerhouses because of their ability to generate ATP by oxidative phosphorylation. They have other functions such as, redox status, reactive oxygen species (ROS) production and regulation of cellular apoptosis [125]. They are the only intracellular organelle apart from the nucleus to contain DNA. During ATP synthesis, electron is transferred from complex I to complex IV of the respiratory chain, which leads to pumping of protons from the mitochondrial matrix into the intermembrane space [126]. Much of the cellular ROS is derived from the mitochondria, mostly from complex I and complex III and enzymes including the alpha-ketoglutarate dehydrogenase complex and those participating in fatty acid beta-oxidation [127–130]. Production of oxygen free radicals is more efficient by reverse electron transfer dependent on succinate (through complex I to NAD^+^) than forward electron transfer with NADH [131]. This reverse electron transfer is an important mechanism of ROS production in many pathological conditions such as hypertension, playing an important role in the development of atherosclerosis [132]. Mitochondrial ROS can itself increase its own production in a positive feedback loop, which is termed ROS-induced ROS release (RIRR) [133, 134]. This is recognized to play a key role initiating mitochondrial depolarization [134].

**Fig. 1:**
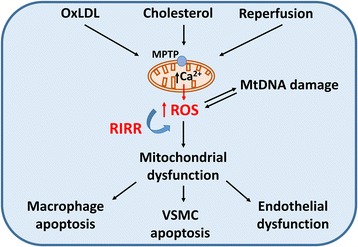
The role of mitochondrial dysfunction in promoting atherosclerosis. OxLDL: oxidized LDL. MtDNA: mitochondrial DNA. ROS: reactive oxygen species. RIRR: ROS-induced ROS release. VSMC: vascular smooth muscle cell

Elevated ROS production can damage a number of macromolecules within the cells, such as proteins, lipids and mitochondrial DNA of different cell types, contributing to atherosclerosis development (Fig. 1). For example, 4-Hydroxynonenal (HNE), an end-product of membrane lipid peroxidation [135], increased ROS production, producing mitochondrial dysfunction and inducing VSMC apoptosis [136]. Reperfusion after ischaemia, which a consequence of atherosclerotic disease, can lead to opening of the mitochondrial permeability transition pore (MPTP) [137, 138]. MPTP opening transiently leads to depolarization of the mitochondrial membrane potential. However, prolonged opening is harmful, as it leads to swelling of the mitochondrial matrix, and rupture of the outer membrane, releasing pro-apoptotic factors to initiate apoptosis [139]. VSMC apoptosis induced by oxidize LDL occurs via two distinct mitochondrial, Ca^2+^-dependent pathways, calpain/mPTP/cytochrome C/caspase-3 and apoptosis-inducing factor [140]. Other cell types such as macrophages also demonstrate mitochondrial dysfunction. Thus, cholesterol loading in macrophages triggers cytochrome c release and activation of caspase-9 and the effector caspases, leading to macrophage apoptosis [141]. Moreover, oxLDL can increase the release of peroxyl radicals, inducing both mitochondrial depolarization, dysfunction and cell lysis in macrophages [142].

There has been a recent interest in the consequences of mitochondrial DNA damage in atherosclerosis. The relationship between mitochondrial DNA damage and oxidative stress has been studied in detail using genetically knockout mice with expressing a proofreading-deficient version of the mitochondrial DNA Polymerase G (POLG) [143, 144]. Mitochondrial DNA damage occurs early in atherogenesis [145]. Mice with double knockout of ApoE and POLG accumulate mitochondrial DNA damage, which promoted atherosclerosis and was associated with the formation of vulnerable plaques [146]. In ApoE-deficient recipients of mutator-mouse bone marrow, fibrous cap thinning and increased necrotic core, which are characteristic of vulnerable plaques, were observed [147]. It is thought that mitochondrial DNA can act as damage-associated molecular patterns (DAMPs), which are recognized by Toll-like receptors (TLRs). TLRs can activate the innate immune system [148].

### Current treatment options and complications

The mainstay treatment options for occlusive vascular disease are stent insertion or bypass grafting. However, significant complications such as stent thrombosis, re-stenosis or vein graft failure can arise [149–151]. There is therefore a need to elucidate the molecular mechanisms underlying these harmful processes in order to devise ways to prevent them. In recent years, the role of oxidative stress, endoplasmic reticulum stress and mitochondrial dysfunction in promoting neointimal proliferation has been intensively studied [152]. VSMCs, the main component of the neointima, are capable of switching from a quiescent and contractile to a proliferative phenotype [153]. The latter is responsible for luminal occlusion. Increased ROS release causes neointimal hyperplasia by promoting VSMC migration and proliferation, as well as collagen deposition within the extracellular matrix [16, 154, 155]. Altered Ca^2+^ signalling plays a critical role in these processes [156, 157]. For example, L-type Ca^2+^ channels are downregulated and transient receptor potential cation (TRPC) channels are upregulated in VSMCs [156]. Of the TRPCs, the Ca^2+^-permeable TRPM2 channel was suggested to be a sensor of oxidative stress [158], and its activation by ROS leads to intracellular Ca^2+^ overload inflammation [159], VSMC, migration, proliferation and apoptosis [158, 160, 161]. Another TRPC isoform, TRPC1, has also been implicated in neointimal proliferation [162].

### Future therapeutic approaches: microRNAs and nanoparticle delivery

The roles of microRNAs in atherosclerosis have been the focus of research in recent years [163]. The use of this model has led to novel therapeutic approaches. MicroRNAs are non-coding RNAs involved in post-transcriptional regulation of genes by RNA silencing [164]. Their generation is under tight control spatially and temporally. Recent work has demonstrated their regulation of flow-dependent vascular remodelling [165]. It appears that some microRNAs, such as miR-10a, miR-19a, and miR-23b are inducible by shear stress and play a protective role in atherosclerosis [166–168]. Other microRNAs, such as MiR-146a and miR-181b, have anti-inflammatory properties by inhibiting the translation of tumor necrosis factor (TNF) receptor-associated factor 6 (TRAF6) and importin α3. This results in inhibition of nuclear factor-kappa B (NF-κB) activity, which is critical in regulating the expression of several chemokines, such as CCL2, CCL5, CCL8, and CXCL9 [166, 169]. In a recent study, polyethylene glycol-polyethyleneimine nanoparticles were used as vectors for microRNA delivery targeting E-selectin of inflamed endothelium of ApoE-deficient mice, which ameliorated endothelial inflammation and atherosclerosis [170]. Phosphatidylserine-based nanoparticles delivering the natural dietary compound curcumin was able to target macrophages and reduce their pro-inflammatory action [171]. Polyethylene glycol-conjugated polyion complex (PIC) vesicles containing drugs were injected into carotid arteries of rats using a balloon catheter, resulting in sustained targeted delivery, providing a proof-of-concept that this approach can be used for treating atherosclerosis in the future [172].

## Conclusions

There is no single ideal animal model for studying a particular clinical condition [173–175]. The general criteria for an appropriate animal model lies are the size, docility, ease of breeding and housing, known genetic profile, analogies with humans and the costs involved. Mice are amenable to genetic modification, allowing identification of genes contributing to the development of atherosclerosis. The understanding on the risk factors and natural history of atherosclerosis offer insights on disease prevention. Identification of the molecular events is important for developing therapeutic strategies to improve endothelial dysfunction, which could slow or even reverse disease progression in atherosclerosis [176, 177].

## Abbreviations

ApoE: ApolipoproteinE
CETP: Cholesteryl ester transfer protein
CHOP: C/EBPα-homologous protein
DAMP: Damage-associated molecular patterns
ER: Endoplasmic reticulum
LDL: Lipoprotein
MPTP: Mitochondrial permeability transition pore
NF-κB: Nuclear factor-kappa B
oxLDL: Oxidized LDL
POLG: Polymerase G
PRR: Pattern recognition receptors
RIRR: ROS-induced ROS release
ROS: Reactive oxygen species
TLR: Toll-like receptor
TNF: Tumor necrosis factor
TRAF6: TNF-associated factor 6
TRPC: Transient receptor potential cation channel
VSMC: Vascular smooth muscle cell
